# Type I Interferon signaling controls the accumulation and transcriptomes of monocytes in the aged lung

**DOI:** 10.1111/acel.13470

**Published:** 2021-09-21

**Authors:** Shanti S. D’Souza, Yuanyue Zhang, Jacob T. Bailey, Ivan T. H. Fung, Marcy L. Kuentzel, Sridar V. Chittur, Qi Yang

**Affiliations:** ^1^ Department of Immunology and Microbial Disease Albany Medical College Albany NY USA; ^2^ Center for Functional Genomics University at Albany‐SUNY Rensselaer NY USA; ^3^ Present address: Rutgers Robert Wood Johnson Medical School Child Health Institute of New Jersey New Brunswick NJ USA

**Keywords:** aging, interferon‐stimulated genes, lung, monocytes, type 1 interferon

## Abstract

Aging is paradoxically associated with a deteriorated immune defense (immunosenescence) and increased basal levels of tissue inflammation (inflammaging). The lung is particularly sensitive to the effects of aging. The immune cell mechanisms underlying physiological lung aging remain poorly understood. Here we reveal that aging leads to increased interferon signaling and elevated concentrations of chemokines in the lung, which is associated with infiltration of monocytes into the lung parenchyma. scRNA‐seq identified a novel Type‐1 interferon signaling dependent monocyte subset (MO‐ifn) that upregulated IFNAR1 expression and exhibited greater transcriptomal changes with aging than the other monocytes. Blockade of type‐1 interferon signaling by treatment with anti‐IFNAR1 neutralizing antibodies rapidly ablated MO‐ifn cells. Treatment with anti‐IFNAR1 antibodies also reduced airway chemokine concentrations and repressed the accumulation of the overall monocyte population in the parenchyma of the aged lung. Together, our work suggests that physiological aging is associated with increased basal level of airway monocyte infiltration and inflammation in part due to elevated type‐1 interferon signaling.

## Graphical abstract

Aging is associated increased Type I IFN signaling which directly and indirectly affects monocyte accumulation and inflammation in the lung.

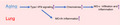

## 1 INTRODUCTION

Aging is a complicated process involving the progressive loss of physiological functions in multiple systems (Boe et al., 2017; Bowdish, 2019; Lopez‐Otin et al., 2013). The lung, a vital organ for gas exchange, is highly susceptible to infections, cancers, and inflammatory disorders with aging (Bowdish, 2019; Lowery et al., 2013; McElhaney et al., 2020; Wang et al., 2020). The lung harbors numerous tissue‐resident and migrating lymphoid and myeloid immune cell subsets. Aging is associated with both deterioration of immune defenses (immunosenescence) and low‐grade chronic tissue inflammation (inflammaging) (Angelidis et al., 2019; Boe et al., 2017; Bowdish, 2019; D'Souza et al., 2019; Franceschi et al., 2000). Mechanisms underlying inflammaging in physiological aging are yet to be better elucidated.

Monocytes are generally considered an important component of host defense and tissue inflammation (De Maeyer & Chambers, 2021). In addition to their capacity to differentiate into phagocytes and dendritic cells, monocytes might be important sources of proinflammatory cytokines and by themselves possess the capability to present antigen (Bassler et al., 2019; Jakubzick et al., 2017). Much of our current knowledge about monocyte aging are from data with human blood samples (Albright et al., 2016; Alvarez‐Rodriguez et al., 2012; De Maeyer & Chambers, 2021; De Martinis et al., 2004; Reynolds et al., 2014; Seidler et al., 2010). Aging‐associated changes in human blood monocytes include decreased antigen processing and presentation activity, reduced metabolic fitness, and altered responses to pattern recognition receptor (Alvarez‐Rodriguez et al., 2012; De Martinis et al., 2004; Hearps et al., 2012; Merino et al., 2011; Nyugen et al., 2010; Pence & Yarbro, 2018, 2019; Roubenoff et al., 1998; Saare et al., 2020; Seidler et al., 2010). In addition, Ly6C^+^ monocytes are increased in the bone marrow and blood of aged mice, which might contribute to increased serum levels of IL‐6 and TNF with aging (Puchta et al., 2016; Strohacker et al., 2012). Whether and how aging might affect monocyte homeostasis in mucosal tissues such as the lung, however, remain incompletely understood.

Interferons (IFN) are important drivers of immune defenses. IFNs are best known for their pivotal role in anti‐viral responses (Makris et al., 2017; McNab et al., 2015; Platanias, 2005). Viral infections induce activation of IFN signaling which stimulates expression of interferon‐stimulated genes with anti‐viral functions (Makris et al., 2017; McNab et al., 2015; Schneider et al., 2014; Schoggins, 2014). IFNs play important roles in initiating inflammatory responses during airway infections and inflammatory disorders (Goritzka et al., 2014, 2015; Lee et al., 2017; Lin et al., 2014; Stetson & Medzhitov, 2006). Despite its potent role in host defense, activation of IFN signaling is also associated with collateral tissue damage and susceptibility to tissue inflammation (Baruch et al., 2014). Previous reports indicate that Type 1 interferon may control monocyte abundance and activity in systemic and tissue infection and inflammation (Channappanavar et al., 2016; Lee et al., 2009, 2017; Seo et al., 2011). The role of Type 1 IFN signaling in the aging of homeostatic lungs, however, remains largely unknown.

In this study, we examined the effects of physiological aging on the abundance and gene expression profiles of monocytes in lungs during homeostasis, as well as the role of IFNs in controlling monocyte abundance and transcriptomes in the aged lung. We reveal that aging leads to monocyte infiltration into the lung parenchyma, which is associated with elevated concentrations of chemokines and a global increase in IFN signaling in the lung. A novel subset of monocytes expressed very high levels of interferon‐stimulated genes, exhibited the greatest transcriptomal changes with aging, and was nearly ablated by in vivo blockade of type 1 interferon. Blockade of Type 1 interferon signaling also reduced the concentrations of chemokines and repressed the accumulation of the overall monocyte population in the lung parenchyma of aged mice. Together, our data suggest that monocyte infiltration and inflammation is a hallmark of physiological lung aging and that type‐1 IFN might control the abundance and transcriptomes of monocytes in the aged lung via multiple layers of direct and indirect mechanisms.

## 2 RESULTS

### 2.1 Aging is associated with increased interferon signaling, elevated concentrations of chemokines in the lung

**FIGURE 1:**
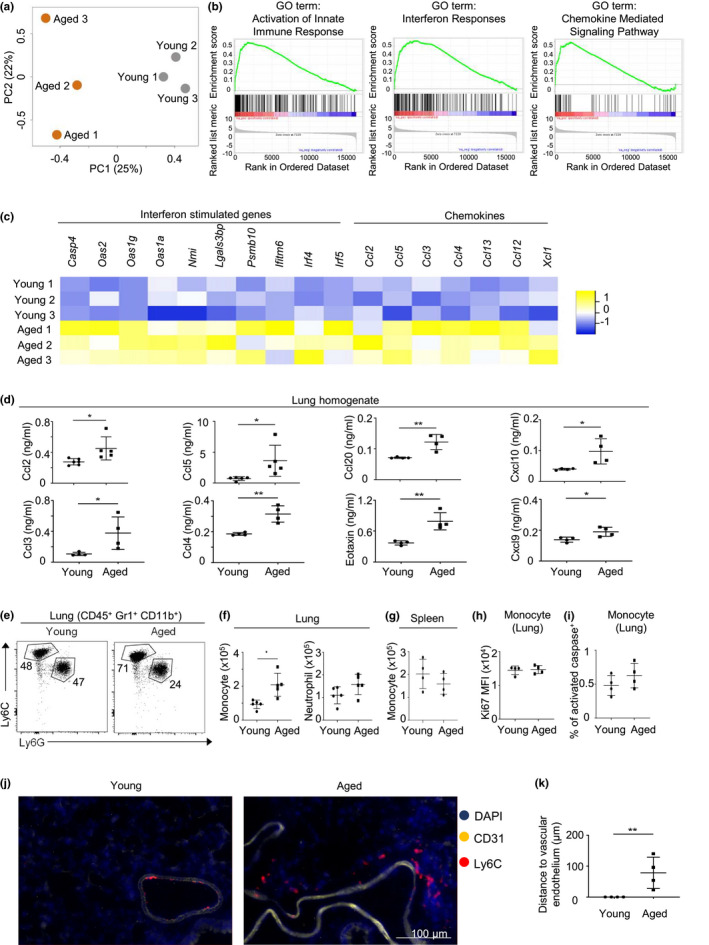
Increased Type I Interferon signaling and chemokines in lungs of aged mice. (a) Principle component analysis of microarray data from total lung cells of young (gray) and aged (orange) female mice. (b) Significant gene sets based on Gene Set Enrichment Analyses (GSEA) of microarray data from lungs of young and aged mice. (c) Heatmap depicting the expression of representative genes in young and aged mouse lungs. *p* value <0.05 for all gene shown in the heatmap. (d) Chemokine concentrations from the lung homogenates of naïve young and aged mice. Data are from 4–5 mice per group. (e) Representative flow cytometry plot of monocytes (CD45^+^ Gr1^+^ CD11b^+^ Ly6C^+^) and neutrophils (CD45^+^ Gr1^+^ CD11b^+^ Ly6G^+^) from lungs of young and aged mice. (f) Number of monocytes and neutrophils in lungs of young and aged mice. (g) Number of monocytes in lungs of young and aged mice. (h) Geometric mean fluorescence intensity of Ki67 staining of monocytes and neutrophils in lungs of young and aged mice. (i) Percentage of activated caspase^+^ monocytes and neutrophils in lungs of young and aged female mice. (j) Representative immunofluorescence staining of monocytes (Ly6C^+^) and vascular endothelial cells (CD31^+^) in the lungs of young and aged mice. (i) Distance between monocytes and vascular endothelium. Data are from female mice (a‐k). Data are from 3 mice per group (a‐c), or are from 4–6 mice per group, two independent experiments (d‐k). Error bars are mean ± SD. *, *p *< 0.05; **, *p *< 0.01

To explore mechanisms of lung aging, we performed genome‐wide microarray analyses of the whole lung tissue from young (2–3 months old) and aged (20–22 months old) mice (Figure 1a). Mice were obtained from NIH via Charles River and rested in the SPF facility of Albany Medical College (AMC) for at least two weeks before experiments. Female mice were housed in regular Allentown cages, with 4–5 mice per cage. Aged male mice exhibited excessive aggressiveness and were individually housed. Female mice were used for the majority of this study due to concerns about potential confounding factors induced by stress and individual housing, but major findings were verified with male mice.

Interestingly, Gene Set Enrichment Analysis (GSEA) revealed that genes that were upregulated with aging were enriched for interferon response genes and chemokines (Figure 1b). Specifically, aging was associated with elevated expression of many interferon‐stimulated genes (ISG) in the lung (Figure 1c). Thus, aging is associated with global increase of interferon signaling in the lung.

The expression of a variety of chemokine genes in the lung was also significantly increased with aging (Figure 1c). Multiplex cytokine assays and ELISAs verified that the concentration of many chemokines, including CCL2, CCL5, CCL20, CXCL10, CCL3, CCL4, Eotaxin, and CXCL9, were extensively increased in the lung homogenate with aging (Figure 1d). Serum chemokine levels were also increased with aging (Figure S1). However, the concentrations of serum chemokines were generally lower than those in the lung (Figure S1). Together, aging is associated with elevated concentrations of chemokines in the lung.

### 2.2 Aging leads to monocyte infiltration in the lung parenchyma

**FIGURE 2:**
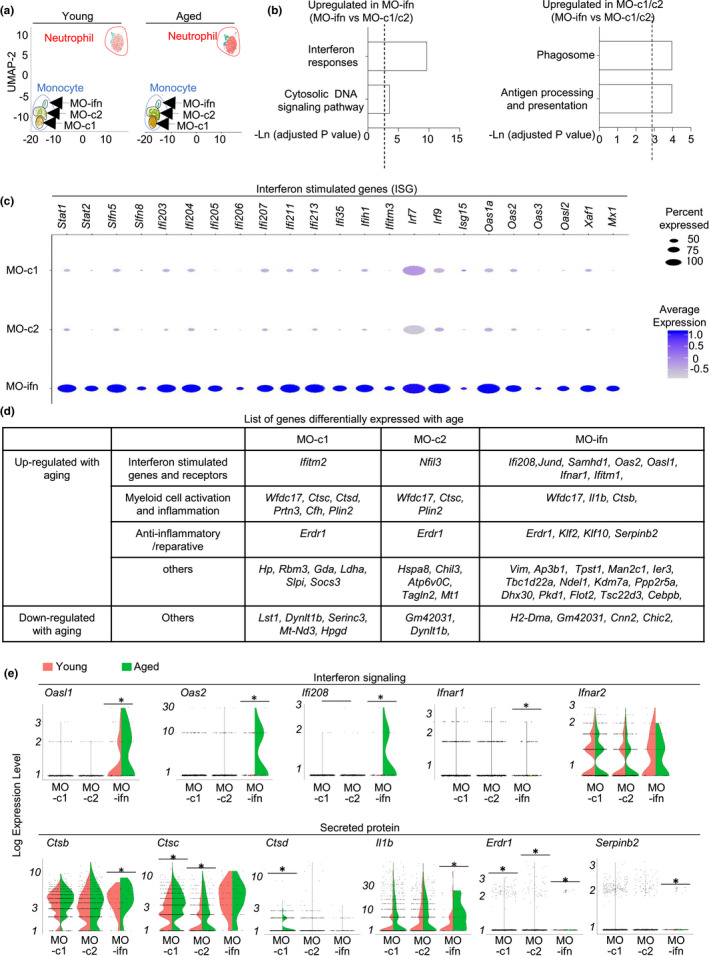
Aging alters the single‐cell transcriptomes of pulmonary monocytes. Single‐cell RNA‐seq were performed with FACS‐sorted Ly6C^+^ monocytes and Ly6G^+^ neutrophils from lungs of young and aged female mice. (a) Unsupervised clustering of cell subsets by UMAP analysis. (b) Pathways of genes highly expressed in MO‐ifn cells, or in MO‐c1 and MO‐c2 cells. *p* value < 0.05, and absolute log (FC) value >0.3 were used as threshold to identify differentially expressed genes. (c) Dot map depicts expressional levels of interferon‐stimulated genes that were highly expressed in MO‐ifn cells. (d) List of genes that were differentiated expressed with aging in each monocyte subset. (e). Violin plots depicts expression of the indicated genes in each monocyte subset in young and aged mice. Data are from 5 mice pooled, per group. * *p* < 0.05

Many of these chemokines have established roles in inducing tissue infiltration of myeloid cells such as monocytes or neutrophils (Charo & Ransohoff, 2006; Ichikawa et al., 2013; Keophiphath et al., 2010; Lin et al., 2014; Schaller et al., 2017; Zhao et al., 2017). We thus examined whether aging is associated increased infiltration of monocytes or neutrophils in the lung. Specifically, we examined the numbers of Ly6C^+^ monocytes and Ly6G^+^ neutrophils, two major subsets of airway infiltrating myeloid cells, in the lungs of young and aged mice. To remove blood cells from lungs, mice were extensively perfused with 50 ml of PBS and the lungs were visibly white before collection. Notably, the numbers of pulmonary Ly6C^+^ monocytes, but not Ly6G^+^ neutrophils, were significantly increased with aging (Figure 2a, b). We did not observe a significant difference in the numbers of monocytes in the spleen between young and aged mice, suggesting that aging‐associated accumulation of monocytes is tissue specific (Figure 2c).

Of note, Ki67 staining and activated caspase assays revealed that monocytes in aged lungs exhibited comparable proliferation and apoptosis rates as those in young mice (Figure 2d, e). Thus, other mechanisms, such as altered cell migration, might contribute to monocyte accumulation in the aged lung.

While the above studies were performed with female mice, we observed similarly increased abundance of monocytes with aging in male mice (Figure S2a). Thus, aging leads to monocyte accumulation in the lung in both sexes.

We next performed immunofluorescence staining to examine the micro‐anatomic locations of monocytes in the lung. In young mice, monocytes were largely restricted to the luminal side of vessels in the lung (Figure 1j, k). Strikingly, the majority of monocytes infiltrated to the lung parenchyma in aged mice (Figure 1j, k). Thus, aging is associated with monocyte infiltration into the lung parenchyma.

### 2.3 scRNA‐seq identified a novel monocyte subset that expressed high levels of interferon‐stimulated genes and exhibited greatest transcriptomal changes with aging

We then performed single‐cell RNA sequencing (scRNA‐seq) to examine the effects of aging on the gene expression profiles of pulmonary Ly6C^hi^ monocytes and Ly6G^+^ neutrophils (Figure S3a). scRNA‐seq detected 25 genes that were differentially expressed in pulmonary monocytes between young and aged mice, and 17 genes that were differentially expressed in neutrophils with age (Figure S3b). Of note, the majority (22/25) of differentially expressed genes (DEGs) in monocytes was upregulated with aging (Figure S3b). The genes upregulated with aging in monocytes were enriched for genes associated with monocyte activation and inflammation. They include the monocyte activation marker *Wfdc17*, proinflammatory cytotoxic molecules *Ctsc* and *Ctsd*, proinflammatory cytokine *Il1b* and other proinflammatory secreted proteins *Prtn3*, *Plin2* and *Cfh* (Figure S3c, d) (Calippe et al., 2017; Dey et al., 2018; Dinarello, 1996; Hamon et al., 2016; Karlstetter et al., 2010; Kessenbrock et al., 2008; Korkmaz et al., 2018; Norman et al., 2018). The few genes downregulated with aging in monocytes have no previously reported roles in tissue inflammation or monocyte activation (Figure S3c, d). Thus, aging might be associated with enhanced proinflammatory gene expression profile in pulmonary monocytes.

We next examined the heterogeneity of pulmonary monocytes. UMAP analysis divided pulmonary monocytes into three subsets, termed MO‐c1, MO‐c2, and MO‐ifn here. MO‐c1 and MO‐c2 cells were transcriptionally similar to each other, whereas MO‐ifn cells exhibited a distinctive transcriptional profile (Figure 2a). MO‐ifn cells expressed high levels of interferon response genes and cytosolic DNA signaling pathway genes, while MO‐c1 and MO‐c2 cells expressed high levels of antigen processing genes and phagosome genes (Figure 2b). In particular, MO‐ifn cells expressed high levels of many ISGs, suggesting that MO‐ifn cells were exposed to high levels of interferon signals (Figure 2c). The list of ISGs highly expressed in MO‐ifn cells included interferon activable proteins (*Ifi203*, *Ifi204*, *Ifi205*, *Ifi206*, *Ifi207*, *Ifi211*, *Ifi213*, *Ifi35*), interferon‐induced helicase C domain (*Ifih1*), interferon‐induced transmembrane protein 3 (*Ifitm3*), interferon activated transcription factors (*Irf7*, *Irf9*, *Stat1*, *Sta2*), oligoadenylate synthases (*Oas2*, *Oas3*, *Oasl2*), Schlafen Family Member 5 (*Slfn5*, *Slfn8*), and other canonical interferon‐stimulated genes (*Xaf1*, *Mx1*) (Figure 2c). Interestingly, MO‐ifn cells, but not MO‐c1/c2 cells, upregulated *Ifnar1* with aging (Figure 2d, e). Many ISGs were also upregulated with aging in MO‐ifn cells, suggesting that aging is associated with further elevated interferon signaling in MO‐ifn cells (Figure 2d, e). More DEGs were identified in MO‐ifn cells than in MO‐c1 and MO‐c2 cells, indicating that the MO‐ifn cells had greater transcriptomal changes with aging than the other monocyte subsets (Figure 2d). Most aging‐induced DEGs in MO‐ifn were upregulated with aging (Figure 2d, e). Interestingly, aging was associated with upregulated expression of both proinflammatory molecules such as *Il1b*, *Ctsb*, and also anti‐inflammatory molecules such as *Erdr1*, *Serpinb2*, in MO‐ifn cells (Figure 2d, e). Both positive regulator of myeloid cell activation such as *Wfdc17*, and negative regulators of monocyte inflammation such as *Klf2* and *Klf10*, were upregulated with aging in MO‐ifn cells (Figure 2d, e). Thus, aging is associated with complicated multi‐faceted molecular changes in MO‐ifn cells.

Of note, MO‐c1 and MO‐c2 cells, which constitute the majority of monocytes in the lung, expressed much lower levels of ISGs than MO‐ifn cells (Figure 2c). MO‐c1 and MO‐c2 did not upregulate expression of IFNARs with aging (Figure 2d, e). MO‐c1 and MO‐c2 cells also did not exhibit extensive upregulation of ISGs expression with aging, indicating that cell intrinsic interferon signaling in these cells might not significantly alter with aging (Figure 2d, e). MO‐c1 and MO‐c2 demonstrated upregulated expression of some proinflammatory and anti‐inflammatory effector molecules such as *Ctsc* and *Erdr1*, but aging‐induced gene expression changes appeared to be fewer in these cells compared to those in MO‐ifn cells (Figure 2d).

### 2.4 Blockade of type 1 Interferon signaling reduced monocyte accumulation in the lung parenchyma of aged mice

**FIGURE 3:**
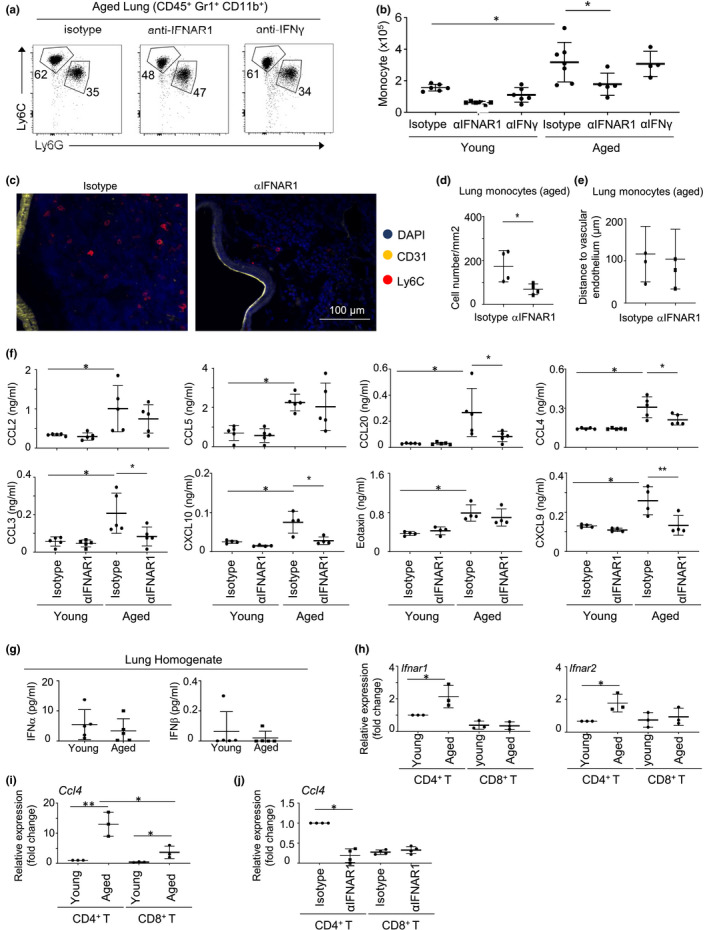
Type I Interferon, but not IFNγ promote monocyte accumulation in the aged lung. Young and aged female mice were treated with either Isotype, anti‐IFNAR1 or anti‐IFNγ antibodies for 1 week. (a) Representative flow cytometry plots showing lung monocytes (CD45^+^ Gr1^+^ Ly6G^−^ Ly6C^+^ CD11b^+^) and neutrophils (CD45^+^ Gr1^+^ CD11b^+^ Ly6C^−^ Ly6G^+^). (b) Total number of lung monocytes from flow cytometry analysis. (c) Immunofluorescence staining of monocytes and vascular endothelial cells in the lungs of aged mice treated with isotype control or anti‐IFNAR1 antibody. (d) Average numbers of monocytes by immunofluorescence staining. (e) Distance between monocytes and vascular endothelium. (f) Chemokine concentrations from the lung homogenates of naïve young and aged mice treated with Isotype or anti‐IFNAR1 antibody for one week. (g) Levels of IFNα and IFNβ in the lung homogenate. (h) CD4^+^ and CD8^+^ T cells were sorted from the lungs of young and aged mice by fluorescence activated cell sorting (FACS). QPCR analyses were performed to examine the expression of *Ifnar1* and *Ifnar2*. (i) Expressional levels of *Ccl4* in FACS‐sorted CD4^+^ and CD8^+^ T cells. (j) Expressional levels of *Ccl4* in CD4^+^ and CD8^+^ T cells from the lungs of aged mice treated with isotype control or anti‐IFNAR1 antibody. Data are from 4–6 mice per group, two independent experiments. Error bars are mean ± SD. *, *p*<0.05; **, *p*<0.01

We next examined whether increased interferon signaling contributed to aging‐associated monocyte changes in the aged lung. We treated aged mice with anti‐IFNAR1 or anti‐IFNγ neutralizing antibodies every other day for 1 week. Anti‐IFNAR1 blocks Type1 interferon signaling, whereas anti‐IFNγ blocks IFNγ signaling. Notably, treatment with anti‐IFNAR1, but not anti‐IFNγ, repressed monocyte accumulation in the aged lung (Figure 3a, b). Neutrophil numbers remained unaffected by anti‐IFNAR1 or anti‐IFNγ treatment (Figure S4a). Notably, the number of pulmonary monocytes exhibited a decreasing trend as early as 24 hrs after a single dose of anti‐IFNAR1, and the decrease became more evident after 1 week of treatment (Figure S4b). Of note, anti‐IFNAR1 treatment reduced monocyte accumulation in the lung parenchyma, but did not significantly alter the micro‐anatomic location of monocytes (Figure 3c‐e). While the above experiments were performed with female mice, we observed that anti‐IFNAR1 treatment similarly reduced monocyte numbers in the lung of aged male mice (Figure S2b). Together, Type 1 interferon signaling promotes monocyte accumulation in the lung parenchyma.

Anti‐IFNAR1 treatment reduced the concentration of multiple chemokines, including CCL20, CCL3, CCL4, CXCL9, and CXCL10, in the aged lung (Figure 3f). Because many of these chemokines have known function in recruiting monocytes, their decreased concentrations may underlie the inhibition of monocyte accumulation by anti‐IFNAR1 (Charo & Ransohoff, 2006; Ichikawa et al., 2013; Keophiphath et al., 2010; Schaller et al., 2017; Zhao et al., 2017).

We next explored the mechanisms that contribute to enhanced interferon signaling in the aged lung. The concentrations of IFNα and IFNβ in the lung were not significantly increased with age, suggesting that other mechanisms might lead to increased interferon signaling in the aged lung (Figure 3g). Because T cells are a significant source of chemokines such as CCL4, we examined whether aging might lead to increased expression of type‐1 interferon receptors in pulmonary T cells (Castellino et al., 2006). Notably, mRNA expression of both *Ifnar1* and *Ifnar2* increased in lung CD4^+^ cells, but not CD8^+^ T cells, with aging (Figure 3h). Pulmonary CD4^+^ T cells dramatically upregulated *Ccl4* expression with age (Figure 3i). Pulmonary CD8^+^ T cells also upregulated *Ccl4* expression with age; however, the expression of *Ccl4* was much higher in CD4^+^ cells than in CD8^+^ T cells in aged mice (Figure 3i). Treatment with anti‐IFNAR1 markedly reduced the expression of *Ccl4* in CD4^+^ cells, but not in CD8^+^ T cells in the aged lung (Figure 3j). Together, our results suggest a model in which aging results in increased expression of type 1 interferon receptors that enhances chemokine production by pulmonary CD4^+^ T cells, which may in turns promote monocyte infiltration in the lung.

### 2.5 The effects of type 1 Interferon signaling blockade on the transcriptomes of monocytes in the aged lung

**FIGURE 4:**
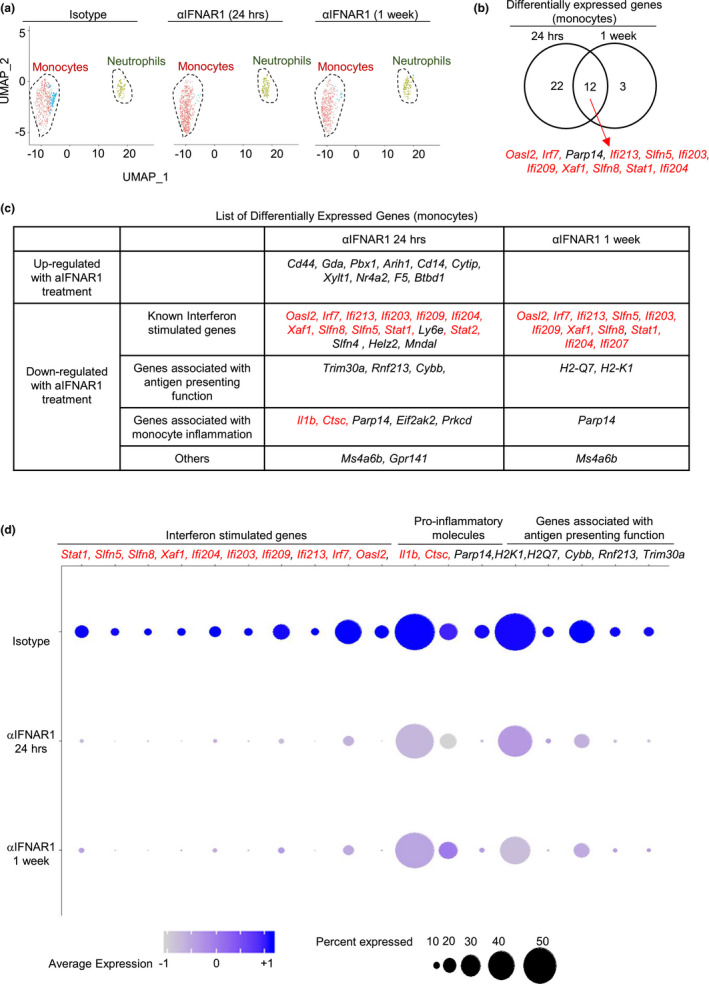
Neutralizing anti‐IFNAR1 treatment alters the transcriptome of aged lung monocytes. (a) Ly6C^+^ monocytes and Ly6G^+^ neutrophils from lungs of aged mice treated with isotype or anti‐IFNAR1 antibody were sorted, and single‐cell RNA sequencing was carried out. UMAP plot reveals two populations of cells. (b) Venn diagram showing the overlap between genes either upregulated or downregulated with one day or one week of anti‐IFNAR1 treatment in aged mouse monocytes. *p* value < 0.05, and absolute log (FC) value >0.3 were used as threshold to identify differentially expressed genes. Genes in red colors were highly expressed in MO‐ifn cells. (c) List of genes that were upregulated or downregulated with one day or one week of anti‐IFNAR1 treatment in aged mouse monocytes. Gene in red colors were genes that were highly expressed in MO‐ifn cells. (d) Dot plots showing representative differentially expressed genes. Gene in red colors were highly expressed in MO‐ifn cells. Data are from 5 mice pooled, per group

We examined whether neutralization of Type 1 interferon may alter the gene expression profile of monocytes in the aged lung. We treated aged mice with anti‐IFNAR1 antibody and performed scRNA‐seq with FACS‐sorted Ly6C^+^ monocytes and Ly6G^+^ neutrophils after 24 hrs or 1 week of treatment (Figure 4a). We used the anti‐IFNAR1 antibody clone MAR1‐5A3, because previous work establishes that MR1‐5A3 specifically neutralizes IFNAR1 bioactivity without inducing antibody‐dependent cellular cytotoxicity (ADCC) effects (Channappanavar et al., 2016; Diamond et al., 2011; Sheehan et al., 2006). We included the time point of 24 hours post‐treatment here, because cells most sensitive to anti‐IFNAR1 treatment were likely absent after 1 week of treatment. Indeed, more DEGS were identified in monocytes after 24 hours of anti‐IFNAR1 antibody compared to 1 week of treatment (Figure 4b). The expression of the majority of DEGS was downregulated following anti‐IFNAR1 treatment (Figure 4c). These genes were enriched for known ISGs, indicating that anti‐IFNAR1 treatment efficiently repressed interferon signaling in pulmonary monocytes of aged mice (Figure 4c). Anti‐IFNAR1 treatment also rapidly repressed the expression of two aging‐associated proinflammatory genes *Ctsc* and *Il1b* in monocytes at 24 hours post‐treatment (Figure 4d). The expression of a few other proinflammatory genes such as *Parp14* was also diminished by anti‐IFNAR1 treatment. QPCR analyses verified that neutralization of IFNAR1 bioactivity led to reduced expression of ISGs and the proinflammatory gene Il1b in the overall monocyte population in the lung (Figure S5). We observed that anti‐IFNAR1 treatment also led to significant reduction of multiple genes involved with antigen presentation function, including *Trim30a*, *Rnf213*, *Cybb*, and MHC molecules, in monocytes of aged lungs (Figure 4d). Thus, blockade of Type 1 Interferon signaling reduced the expression of ISGs and altered expression of other effector molecules in monocytes of the aged lung.

**FIGURE 5:**
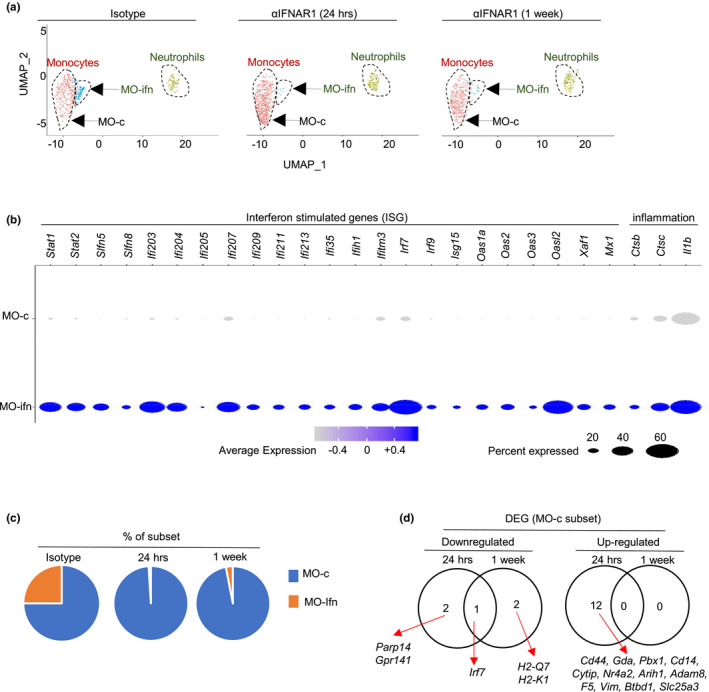
Neutralizing anti‐IFNAR1 treatment differentially alters the transcriptomes of MO‐ifn and MO‐c cells. Single‐cell RNA‐seq (scRNA‐seq) was performed with Ly6C^+^ monocytes and Ly6G^+^ neutrophils sorted from lungs of aged female mice treated with isotype or anti‐IFNAR1 antibody. (a) Unsupervised clustering by UMAP analysis. (b) Dot plots showing genes that were highly expressed in MO‐ifn cells. *p* value < 0.05, and absolute log (FC) value >0.3 were used as threshold to identify differentially expressed genes. (c) Percentages of MO‐ifn and MO‐cells in aged mice treated with isotype or anti‐IFNAR1 antibody. (d) List of genes that were differentially expressed with aging in MO‐c cells. Data are from 5 mice pooled, per group

We next explored the effects of type 1 interferon signaling blockade on the heterogeneity of monocytes in the aged lung. Of note, the scRNA‐seq experiment performed in Figure 2 (experiment 1, comparing cells from young and aged mice) and the experiment performed in Figures 4 and 5 (experiment 2, comparing cells from aged mice treated with isotype and anti‐IFNAR1 antibody) were two independent experiments. Although these two independent experiments might yield different results in clustering cells with similar gene expression profiles, we expected that cell populations with a distinctive transcriptional profile can be consistently identified in both experiments. Indeed, the novel MO‐ifn cells identified in experiment 1 (Figure 2a) again formed a distinctive cell population in the scRNA‐seq data generated from experiment 2 (Figure 5a). Notably, the list of ISGs highly expressed by MO‐ifn cells in both scRNA‐seq datasets largely overlapped, verifying data reproducibility (Figures 2c, 5b). Results from Experiments 2 did not further distinguish MO‐c1 and MO‐c2 cells (Figure 5a). These results were expected because MO‐c1 and MO‐c2 are transcriptomally close to each other (Figure 2a). We thus named the non‐MO‐ifn monocytes MO‐c cells (Figure 5a).

Of note, treatment with anti‐IFNAR1 rapidly ablated MO‐ifn cells within 24 hours, indicating that the maintenance of MO‐ifn depends on type‐1 interferon signaling (Figure 5a, c). We noted that almost all the ISGs that were downregulated in the overall monocyte population by anti‐IFNAR1‐treatment were highly expressed by MO‐ifn cells (genes in red colors in Figure 4 b‐d). Two proinflammatory genes highly expressed by MO‐ifn cells, *Il1b* and *Ctsc*, were also significantly downregulated in the overall monocyte population by anti‐IFNAR‐1 treatment (*Ctsc* and *Il1b*) (Figure 4b‐d, Figure 5b). Thus, the abolishment of MO‐ifn cells might contribute largely to reduced expression of ISGs and proinflammatory genes in the overall monocyte population in anti‐IFNAR1‐treated aged mice.

We next examined the effects of anti‐IFNAR1‐treatment on the transcriptomes of MO‐c cells. MO‐c cells were the major monocyte population in the lung, and expressed much lower levels of ISGs than MO‐ifn cells (Figures 2a, 5a). Notably, we did not observe extensive downregulation of ISG expression in MO‐c cells following anti‐IFNAR1 treatment. The only ISG that was downregulated in MO‐c cells by anti‐IFNAR1 treatment was *Irf7* (Figure 5d). Because *Irf7* expression might also be regulated by other signaling pathways, these data indicate that type‐1 interferon is unlikely to directly act on MO‐c cells. Of note, MO‐c cells are the major monocyte population in the lung (Figures 2a, 5a), and anti‐IFNAR1 treatment leads to significant reduction of the overall monocyte population in the lung (Figure 3a‐d). Thus, type‐1 interferon might influence MO‐c cells via indirect mechanisms such as positive regulation of chemokine expression by T cells (Figure 3f‐K).

### 2.6 The specific chemokines regulated by Type 1 interferon during influenza infection differ from the chemokines regulated by Type 1 interferon in physiological aging

**FIGURE 6:**
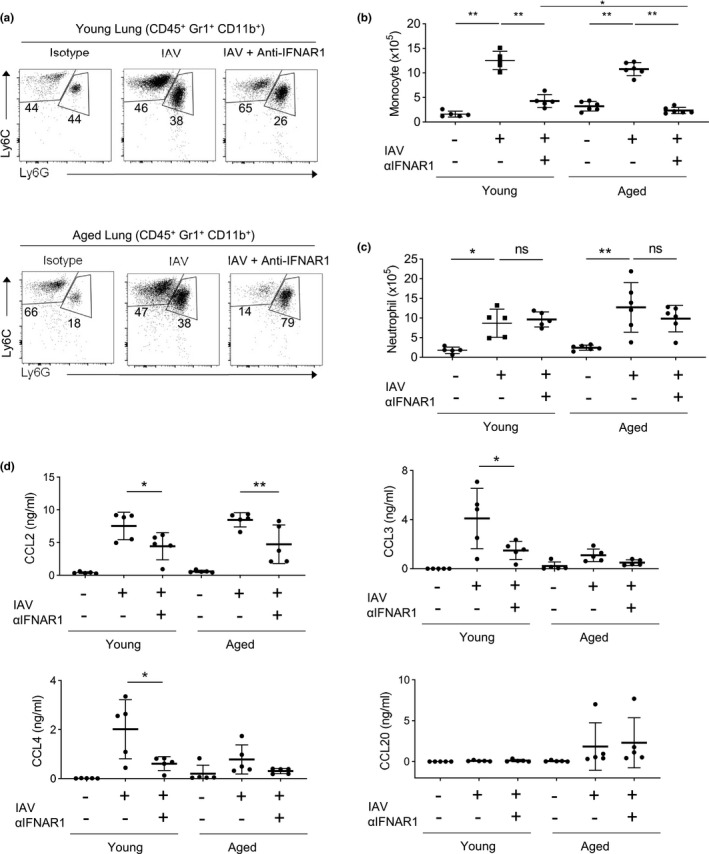
Aging monocytes further expand during Influenza infection in an IFNAR1‐dependent manner. Young and aged mice were either treated with anti‐IFNAR1 and/or infected with IAV and sacrificed on day 6 of infection. (a) Representative flow cytometry plots showing lung monocytes (CD45^+^ Gr1^+^ CD11b^+^ Ly6G^−^ Ly6C^+^) and neutrophils (CD45^+^ Gr1^+^ CD11b^+^ Ly6C^−^ Ly6G^+^). (b) Total number of lung monocytes. (c) Total number of lung neutrophils. (d) Chemokine concentrations from the lung homogenates. Data are from 5–6 mice per group, two independent experiments. Error bars are mean ± SD. *, *p *< 0.05; **, *p *< 0.01

We next examined monocyte responses to influenza infection in aged mice and the specific roles of Type 1 interferon in regulating influenza‐induced monocyte responses in the aged lung. Massive monocyte infiltration was observed in aged mice at day 6 post‐infection of the CA/04 Influenza A Virus (IAV) strain (Figure 6a, b). The numbers of monocytes in aged infected mice were comparable to those in young infected mice (Figure 6a, b). Anti‐IFNAR1 treatment repressed monocyte infiltration in the lungs of IAV‐infected aged mice (Figure 6b). Consistent with previous reports, anti‐IFNAR1 treatment also inhibited monocyte infiltration in the lungs of IAV‐infected young mice (Figure 6b) (Lee et al., 2009; Seo et al., 2011). Of note, anti‐IFNAR1 treatment appeared to induce even greater effects on pulmonary monocytes in IAV‐infected aged mice than those in infected young mice, because the numbers of monocytes in the lungs of anti‐IFNAR1 treated and IAV‐infected aged mice were even lower than those in young mice (Figure 6b). Neutrophils also infiltrated in both young and aged mice during influenza infection, but their numbers were unaltered by anti‐IFNAR1 treatment (Figure 6c).

We then examined chemokine concentrations in IAV‐infected mice. Influenza infection leads to drastically elevated CCL2 concentration in the lungs of both young and aged mice, and anti‐IFNAR1 treatment repressed lung CCL2 concentrations in both young and aged mice (Figure 6d). Of note, CCL2 concentration was also increased with aging at homeostasis in naïve mice, but its concentration was not altered by anti‐IFNAR1 treatment at homeostasis (Figure 3f). In addition, the chemokines that were regulated by Type‐1 interferon at homeostasis, such as CCL3, CCL4 and CCL20, were not significantly changed by anti‐IFNAR1 treatment in IAV‐infected mice (Figure 6d). Thus, although Type 1 interferon promoted both IAV and aging‐induced pulmonary monocyte infiltration, the specific chemokines regulated by Type 1 interferon during influenza infection differ from the chemokines regulated by Type 1 interferon in physiological aging.

Treatment with anti‐IFNAR1 antibodies did not significantly change the survival or weight loss of aged mice in IAV infection (Figure S6). Of note, type‐1 interferon affects many cell types and molecular pathways. Future research with aged transgenic mice that specifically lack interferon signaling in monocytes might be needed, in order to understand the precise role of monocyte‐intrinsic interferon signaling in influencing tissue inflammation and host defense during IAV infection.

## 3 DISCUSSION

Our results together reveal that aging is associated with increased basal levels of monocyte inflammation in the lung partly via Type 1 interferon dependent mechanisms. Our data indicate that type 1 interferon might control the abundance and activity of different monocyte subsets in the lung via distinct mechanisms. Type‐1 interferon might directly promote the maintenance of MO‐ifn cells, a novel monocyte subset that exhibits greatest transcriptional changes with aging. Type‐1 interferon signaling might also indirectly control the abundance of MO‐c cells, in part via regulation of chemokine expression by T cells. Our data also suggest that increased expression of type‐1 interferon receptors, but not elevated concentrations of type‐1 interferons, underlie increased type‐1 interferon signaling in the aged lung. Together, these data provide insights into the complicated multiple layers of cellular and molecular mechanisms underlying physiologic lung aging.

Our two independent scRNA‐seq experiments both identified a novel monocyte subset MO‐ifn. MO‐ifn cells express high levels of ISG, and their maintenance is dependent on type‐1 interferon signaling. MO‐ifn cells upregulate IFNAR expression with aging, and they demonstrate greater gene expression changes with aging than the other monocytes. The mechanisms by which MO‐ifn cells possess high levels of cell intrinsic interferon signaling remain unknown. Because MO‐ifn cells did not express higher levels of IFNARs than MO‐c cells, we predict that MO‐ifn cells might reside in a special microenvironmental niche with high concentrations of type‐1 interferon. In future efforts, it would be highly worthwhile to explore such potential microenvironmental niche and the other immune and non‐immune cells that MO‐ifn cells interact within this niche.

Our data also reveal surprising characteristics of MO‐c cells, the major monocyte subset in the lung. Notably, MO‐c cells express low levels of ISG, and the expression of ISGSin MO‐c cells is largely intact following anti‐IFNAR1 treatment. Thus, cell intrinsic type‐1 interferon signaling is unlikely to play a significant role in controlling MO‐c cell activity. However, blockade of type‐1 interferon repressed the accumulation of the overall monocyte population in the lung parenchyma of aged mice, suggesting that type‐1 interferon might promote the abundance of pulmonary MO‐c cells via indirect mechanisms. Indeed, aging leads to increased concentrations of many chemokines whose levels are reduced by anti‐IFNAR1 treatment. Thus, type‐1 interferon may indirectly promote the accumulation of MO‐c cells in the aged lung via mechanisms such as inducing airway chemokine expression and release. These data together indicate that the role of type‐1 interferon in controlling tissue inflammation and aging is complicated and multiple‐faceted. In future research, it would be interesting to fully dissect the complete molecular and cellular network that is controlled by type‐1 interferon and other key inflammatory and regulatory signals in the aging context.

Our work also indicates that increased IFNAR1 expression, but not increased concentrations of IFNAR, might underlie enhanced IFNAR signaling in the aged lung. The mechanisms by which aging induces increased IFNAR expression in certain cells subsets remain an intriguing enigma. Both cell intrinsic and microenvironmental changes might contribute to the increased IFNAR expression with aging. Future efforts to dissect these mechanisms might provide important insights into the mechanisms of mammalian aging.

### 3.1 Experimental Procedures

#### 3.1.1 Mice

Young (2–3 month) and aged (20–22 month) female C57BL/6 mice were obtained from the National Institute of Aging via Charles River. All animal experiments were performed according to protocols approved by the Institutional Animal Care and Use Committee at Albany Medical Center.

#### 3.1.2 Antibody treatments

Antibodies were purchased form BioXCell. For anti‐IFNAR1 (Clone MAR1‐5A3) and anti‐IFNγ (Clone XMG1.2) treatment, mice were treated with intraperitoneal (i.p.) injections once, or every other day for a week (for a total of 4 injections) with 500μg of antibody. Control mice were injected i.p. with Isotype control antibody (Clone C1.18.1). For anti‐PDCA1 treatment, mice were treated with anti‐PDCA1 antibody (Clone 927) or Isotype control antibody (Clone LTF‐2) via i.p. injections every other day for a week (for a total of 4 injections) with 500μg of antibody. Mice were sacrificed the day after the last treatment.

#### 3.1.3 Isolation of hematopoietic cells from lungs and spleen

Mice were perfused with 50ml cold PBS, and the lungs were visibly white before collection. Tissues were minced using scissors and digested in Hank's balanced salt solution with 0.2 mg/ml Liberase TM (Roche) and 0.1 mg/ml DNAse1 (Roche) for 30 min at 37℃. Cells were strained through a 70 μM cell strainer to obtain single cells.

#### 3.1.4 Flow cytometry

Antibodies were purchased from Biolegend or eBioscience. Antibodies used for flow cytometry included anti‐CD45.2 (104), anti‐CD11b (M1/70), anti‐Gr1 (Gr‐1), anti‐Ly6C (AL‐21), anti‐Ly6G (1A8), anti‐B220 (RA3‐6B2), anti‐CD11c (N418), anti‐PDCA1 (eBio927). DAPI or PI were used as viability dyes to exclude dead cells. Flow cytometric analysis was performed using FACSCanto (BD) and data were analyzed using FlowJo. Fluorescence activated cell sorting (FACS) were performed with a FACSAria sorter.

Activated caspase assays were performed with CellEvent™ Caspase‐3/7 green flow cytometry assay kit (Thermo Fisher Scientific), according to the manufacturer's instruction. Specifically, cells were incubated with CellEvent™ Caspase‐3/7 Green Detection Reagent that contained caspase 3/7 recognition sequence encoded in a four‐amino acid peptide at 37 C for 30 mins. Cells were examined by flow cytometry analysis following the manufacturer's instructions.

#### 3.1.5 Chemokine and cytokine measurement

Lungs were homogenized in 1 ml cold PBS, and supernatant was used for chemokine measurement. IFNa and IFNb levels were measured using ProQuantum High Sensitivity Immunoassays (Thermo Fisher Scientific), according to the manufacturer's instructions. CCL2 concentration was measured using CCL2 Mouse Uncoated ELISA kit (Thermo Fisher Scientific), following manufacturer's instructions. All other chemokines were examined using the LegendPlex Mouse Proinflammatory Chemokine Panel (Biolegend).

#### 3.1.6 Immunofluorescence staining

For immunofluorescence staining, mice were perfused with 50 ml of PBS followed by 50 ml of 4% paraformaldehyde. Lungs were fixed in 4% paraformaldehyde overnight at 4oC, transferred to 30% sucrose until sinks, embedded in OCT in −80oC. Slices were sectioned using a CM1850 cryostat (Leica). Sections were first stained with anti‐Ly6C (Abcam, clone ER‐MP20) and Goat‐anti‐Rat IgG (H+L) A555 secondary antibody. Sections were then stained with anti‐CD31 A488 (eBioscience, Clone 390) antibody. Slides were mounted with antifade with DAPI, and imaged using an Axio Observer fluorescence microscope (Zeiss).

#### 3.1.7 Single‐cell RNA‐sequencing and microarray analysis

For single‐cell RNA sequencing, monocytes and neutrophils of young and aged mice, Ly6C^+^ monocytes and Ly6G^+^ neutrophils were sorted from lungs of mice using TotalSeq C0951 antibody (Biolegend) to label cells from young mice or TotalSeq‐C0952 (Biolegend) to label cells from aged mice. For scRNA‐seq of monocytes and neutrophils from aged mice treated with anti‐IFNAR1, aged mice were treated with isotype control once or with anti‐IFNAR1 antibody (500 μg, intraperitoneally) once or every other day for a week (a total of 4 treatments). Mice were sacrificed the day after the last injection. Ly6C^+^ monocytes and Ly6G^+^ neutrophils were sorted by FACS. scRNA‐seq was performed as we previously described (Fung et al., 2020; Harly et al., 2019; Zhang et al., 2020). scRNA‐seq libraries were generated using the chromium 5’ single‐cell gene expression kit (10x genomics) according to the manufacturer's instructions. Sequencing was carried out using a NextSeq 500 (Illumina). Preliminary data analysis was carried out using Cellranger V3.0.2. Data were normalized and scaled using Seurat (Stuart et al., 2019). Cells were clustered by Uniform Manifold Approximation and Projection (UMAP) function in Seurat. Differentially expressed genes were identified based on Seurat's normalized data, and a Wilcoxon rank sum test was used to test statistical significance. The scRNA‐seq data have been deposited in the Gene Expression Omnibus under the accession numbers GSE167236 and GSE167170.

For microarray analysis, lung lobes from 3 young and 3 aged mice were resected, and total RNA was purified using TRIzol following the manufacturer's instructions. Microarray was carried out at Boston University Microarray and Sequencing Resource Core Facility. The microarray data have been deposited in the Gene Expression Omnibus under accession number GSE167215.

#### 3.1.8 Influenza infection

For Influenza infection, mice were infected with 340 PFU of Influenza A virus (CA04 strain), administered via the intranasal route. Mice were treated with anti‐IFNAR1 or isotype control antibodies (500 μg i.p.) every other day, starting from the day before infection, up to day 5 post‐infection. Monocyte responses were measured at day 6 post‐infection.

### 3.2 Statistical analysis

Differences between two groups was examined using two‐tailed Student's *t* tests. Differences between more than two groups was analyzed using one‐way ANOVA. *p*<0.05 was considered significant. Wilcoxon rank sum test was used to test the statistical significance of differentially expressed genes by scRNA‐seq.

## CONFLICT OF INTEREST

The authors declare no competing financial interests.

## AUTHOR CONTRIBUTIONS

S. D'Souza performed most of the experiments, with the help of Y. Zhang, J. T. Bailey, I. Fung, S Chittur, M. Kuentzel, and Q. Yang. S. D'Souza and Q. Yang wrote the manuscript. All authors reviewed and approved the manuscript.

## Supporting information

Fig S1‐S6

## Data Availability

The data that support the findings of this study are available in GEO at https://www.ncbi.nlm.nih.gov/geo/ reference number GSE167236 and GSE167170 and GSE167215.
