# Supplementary figures and images for: Type I Interferon signaling controls the accumulation and transcriptomes of monocytes in the aged lung
Source: Aging Cell. 2021 Sep 21;20(10):e13470. doi: 10.1111/acel.13470 (PMC8520712; doi:10.1111/acel.13470)

Figure S1

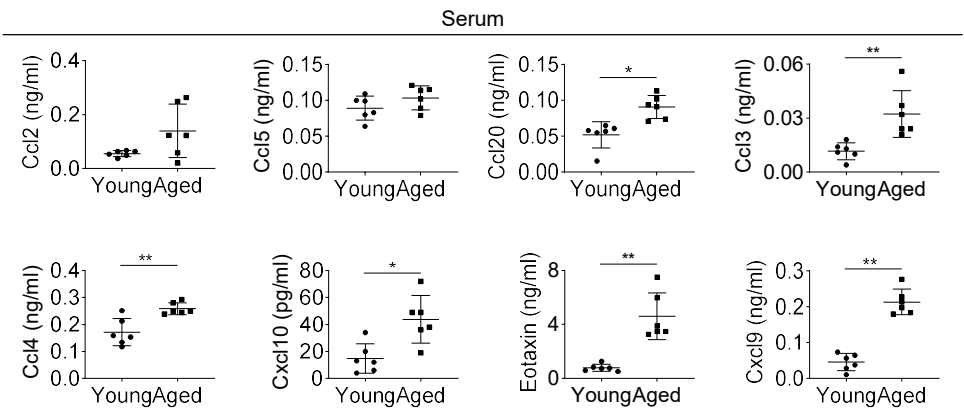

Figure S2

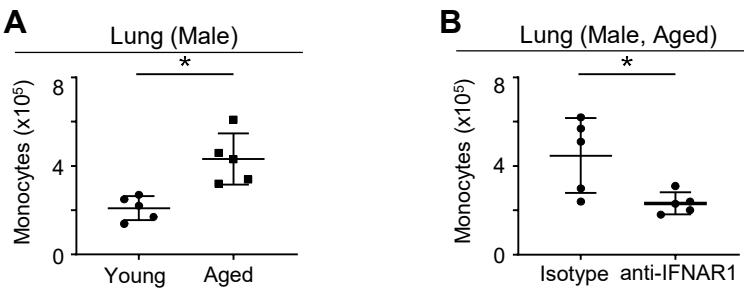

Figure S3

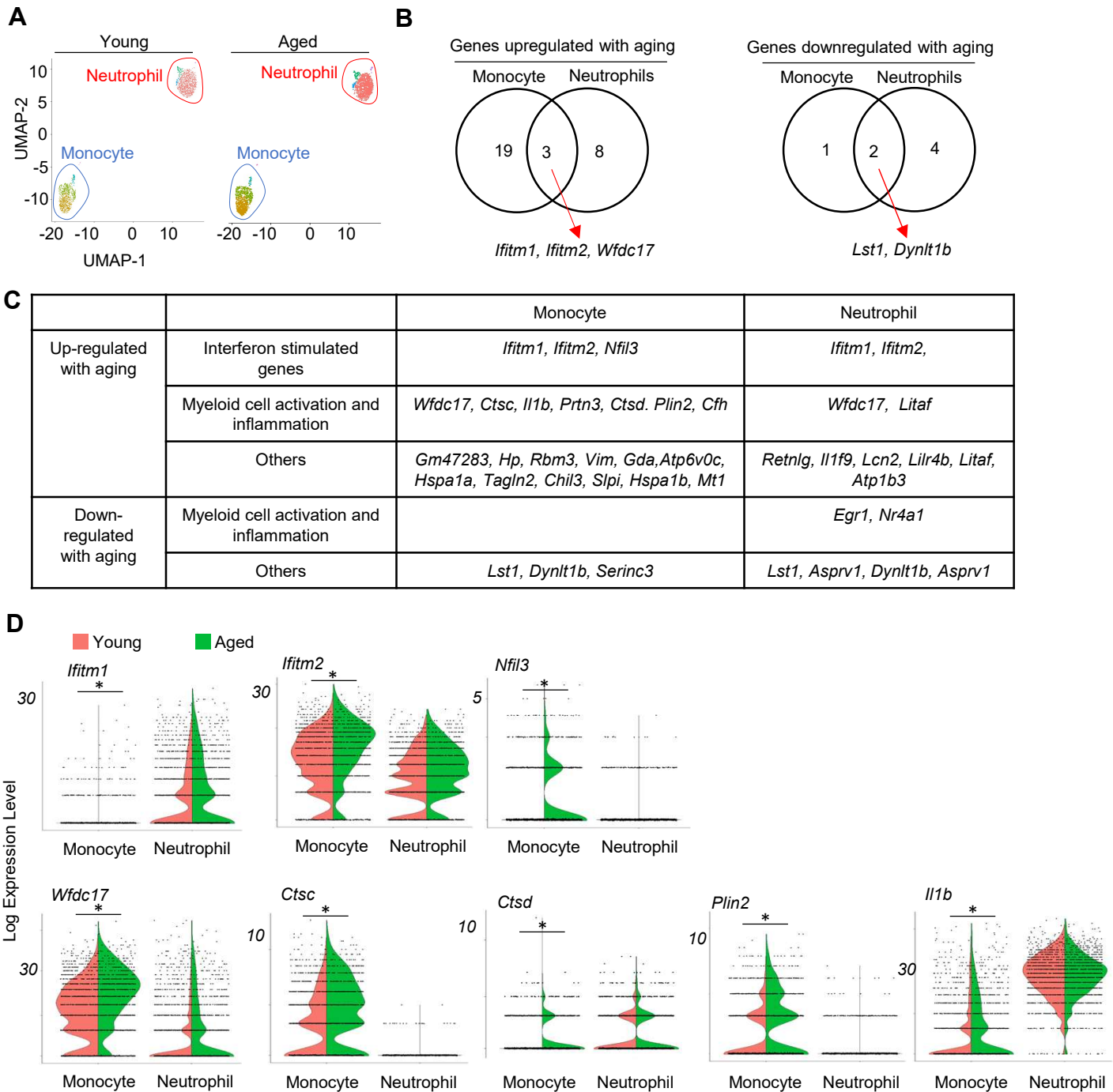

Figure S4

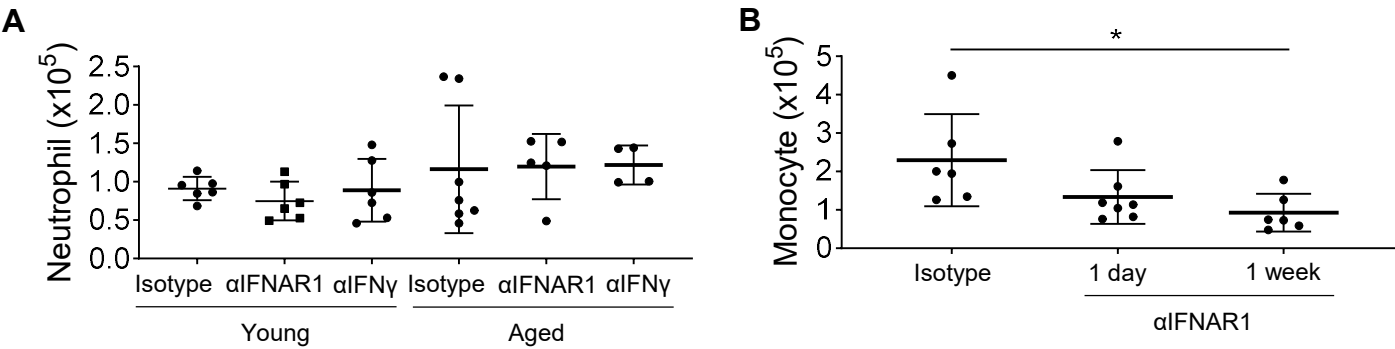

Figure S5

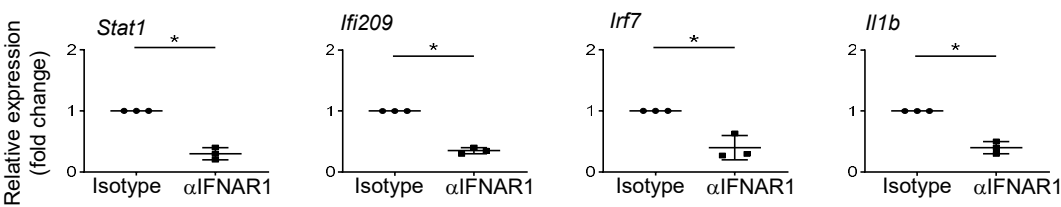

Figure S6

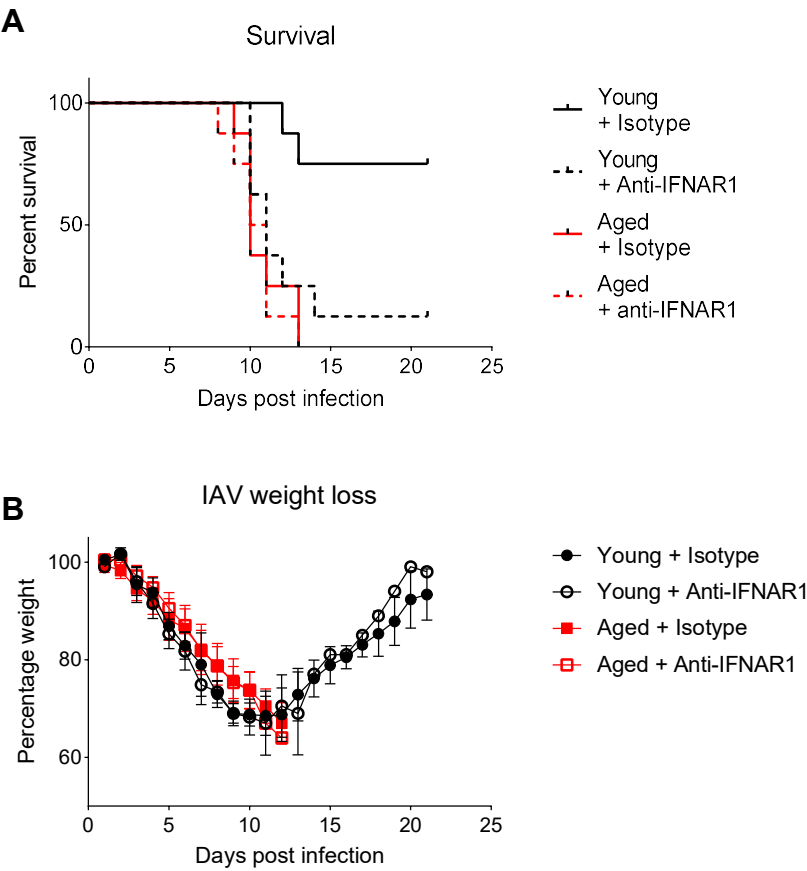

Supplement: Supplementary file 1 — Fig S1‐S6 [file ACEL-20-e13470-s001.pdf]
